# The Association Between Problem Gambling and Suicidal Ideations and Attempts: A Case Control Study in the General Swedish Population

**DOI:** 10.1007/s10899-020-09996-5

**Published:** 2021-01-25

**Authors:** Kristina Sundqvist, Peter Wennberg

**Affiliations:** 1 Department of Psychology, Stockholm University, Stockholm, Sweden; 2 Department of Public Health Sciences, Stockholm University, Stockholm, Sweden; 3 Department of Global Public Health, Karolinska Institutet, Solna, Sweden

## Abstract

The association between problem gambling and suicidal behaviours is well established in treatment seeking populations, but less explored among sub-clinical problem gamblers in the general population. The aim of this study was to examine the association between problem gambling (including moderate risk gambling) and suicidal ideations/suicide attempts, in the general Swedish population. Another aim was to compare problem gamblers with and without suicide ideation/attempts. A case-control study nested in the Swelogs cohort was used. Both ideations and attempts were about twice as frequent among the cases compared to the controls. After controlling for socio-economic status and life-time mental health problems, suicidal ideation, but not attempts, remained significantly higher among the cases compared to the controls. The largest difference between attempters and non-attempters were on payment defaults and illicit drug abuse, whereas depression yielded the largest difference between ideators and non-ideators. Problem gambling severity (PGSI 8+) resulted in the smallest difference, compared to the other variables, between attempters and non-attempters. Even though no conclusion regarding the casual relationship can be drawn in this type of study, it seems like sub-clinical levels of problem gambling might have an impact on suicidal ideations whereas for suicide attempts to occur, other factors need to be present. In addition to mental health issues, financial difficulties may be such factors.

## Introduction

Gambling is a leisure activity that can progress and have severe consequences. Studies worldwide have found prevalence rates of problem gambling ranging from 0.5 to 7.6 pending on study and cultural settings, with an average across all countries of 2.3% (Williams et al. 2012). Problematic gambling has been associated with other psychiatric conditions, criminality and even suicide (Lorains et al. 2011).

There are several terms for excessive gambling behavior. In the Diagnostic and Statistical Manual of Mental Disorders, ‘gambling disorder’ is used to describe severe gambling problems (5th ed; DSM-5; American Psychiatric Association 2013). However, the broader term problem gambling, used in this study, is often used to include those that do not fill the criteria’s for a diagnosis but still suffer significant consequences from their gambling (Blaszczynski and Nower 2002). Suicidal behavior can be thought of as existing on a spectrum (World Health Organization 2014), including self-harm, ideations (thoughts, wishes and plan of taking one’s own life) and actual suicidal attempts. Recent developments in suicide theory and research have proposed the ‘ideation-to-action framework’, which suggests that the development of suicidal ideation and the progression from ideation to suicide attempts are distinct phenomena with diverse etiology (Klonsky et al. 2016).

Previous research on clinical samples has identified a link between problem gambling and both suicidal ideations (thoughts, wishes and plans) and attempts (Moghaddam et al. 2015). As negative consequences and losses grow, suicide may be seen as the only solution to both financial and psychological stress (Hodgins et al. 2006). Studies involving treatment-seeking problem gamblers have shown that 36% to 50% had a history of suicidal ideation (Battersby et al. 2006; Lejoyeux et al. 2002; Petry and Kiluk 2002), and 20% to 30% of problem gamblers had made suicide attempts (Guillou-Landreat et al. 2016; Thompson and Schwer 2005). A register based study in Sweden showed a 15-fold increase in suicide mortality for individuals 20–74 years old diagnosed within the health care system with gambling disorder, compared to the general population (Karlsson and Håkansson 2018). In a case-control study it was found that both suicide attempts and ideations were over 10 times more common among the cases (diagnosed with gambling disorder) compared to their controls (Black et al. 2015). In addition, disorders commonly comorbid with problem gambling, such as mood and substance use disorders are also associated with suicide (Windfuhr and Kapur 2011), thereby further increasing an individual’s vulnerability for suicidal ideation and/or attempts. A recent study found that even though Suicidal behavior were common in patients with problem gambling, it was markedly more common in the presence of other comorbid disorders (Håkansson et al. 2020).

Studies on the relationship between gambling and suicide in the general population are rare. This is unfortunate since treatment seeking samples are unrepresentative due to selection bias and hence differ from problem gamblers in the general population. Studies suggests that clinical samples mainly comprise of white, middle-aged gamblers (Volberg 1994), with more severe problem gambling symptoms and are more likely to have experienced comorbid conditions (Lorains et al. 2011). A study by Hodgins et. al. (2006) revealed that suicide attempts among a community sample of problem gamblers were nearly universally made when participants reported feeling depressed, and that suicidal ideations predated problem gambling onset with about ten years. Additionally, more than half of these respondents reported that the majority of their suicide attempts had been made under the influence of alcohol or other drugs. In another Canadian study, the association between problem gambling and suicide ceased to be statistically significant when additional mental disorders were included in the analysis (Newman and Thompson 2003). It was concluded in this study that the association between gambling and suicide attempts may be due to the common factor of mental illness. However, this contradicts another Canadian study (Newman and Thompson 2007), where suicidality was significantly associated with problem gambling, despite adjustment for other sociodemographic and psychiatric variables.

Studies exploring confounding factors have found problem gambling severity, being in depth, high financial losses, mood disorders, alcohol/substance use disorders and female gender to affect the association between problem gambling and suicidal events (Battersby et al. 2006; Bischof et al. 2016; Manning et al. 2015; Wong et al. 2010). But studies on the roles of those factors are inconclusive. In the study by Bettersby (2006), for example, gender was *not* significantly associated with suicidal events, and in the study by Bischof (2016), gambling severity was *not* independently associated with suicidal events. The same study also found EGM gambling to be associated with suicidal events (Bischof et al. 2016).

As described above, treatment seeking samples most likely differ from community-based samples, for instance in terms of problem gambling severity and other co-occurring psychiatric conditions. The association among problem gambling and suicidality is less explored within community-based samples compared to treatment seeking samples, as well as among sub-clinical samples.

The aim of this study was to examine the association between problem gambling and suicidal ideations, as well as the association between problem gambling and suicide attempts, in the general population.

A second aim was to explore other factors possibly contributing to the associations by comparing problem gamblers with and without suicidal ideations/attempts.

## Methods

### Design

The Swedish longitudinal gambling study (Swelogs), is a research program on gambling and problem gambling in Sweden, initiated in 2008 and managed by the Public Health Agency of Sweden (Romild et al. 2014; The Public Health Agency of Sweden 2013). Swelogs includes two data sets called the Epidemiological track (EP) and the In-Depth (ID) track. The EP-track consisted of a stratified random sample of 15,000 individuals (the procedure is decribed in detail by Romild et al. 2014).The ID-track is a case-control study nested in the Swelogs cohort. Details about the data collection has been previously described (Fröberg 2015; Sundqvist and Rosendahl 2019), and will be described briefly below.

The purpose of the ID track is to collect information about the mental health of the study participants in a lifetime perspective. Caseness was defined by scoring 3 or more on PGSI 12 months or on SOGS-R life (n = 591). The controls (n = 2400) were frequency matched to the cases based on sex and age, with a case/control ratio of 1:3. Two data collections have been carried out within the Swelogs ID track (ID1 and ID2; 2011 and 2013). In addition, a qualitative study was conducted (Samuelsson et al. 2018). Data was collected through telephone interviews conducted by the Centre for Psychiatry Research at Karolinska Institutet, and through postal questionnaires among non-participants in the interview. The interviews covered gambling related issues, a psychiatric diagnostic assessment, life stressors and adverse events, family and participant socio-demographic aspects. Socio-demographic information from official registers was linked to the data set. The measures will be described in more detail in the Methods section.

### Participants

For the present study, participants from ID1 (2011) was used, since life-time measures was not used in ID2. The sample consisted of 427 cases (34% female) and 1583 controls (35% female). See Table 1 for participant characteristics.

**Table 1:** Characteristics of cases and controls. Odds ratios and 95% confidence intervals

	Case % n = 427	Control % n = 1583	OR	95% CI
Matching variables				
Gender F/M	35/65	34/66		
Age M (SD)	28.2 (13.7)	28.1 (14.7)		
Other sample characteristics				
SES (low vs high)				
Low	27	20	1.7	1.2 – 2.2
Medium	44	45		
High	28	35		
Any depression	33.3	20.2	2.0	1.5 – 2.5
Any anxiety	24	15	1.9	1.4 – 2.5
Any alcohol Dependence	34.3	15.9	2.8	2.1 – 3.6
Any Illicit Drug Use	7.7	3.7	2.2	1.3 – 3.5

### Measures

#### Mental Health and Suicidality

Mental health (anxiety, depression and substance use) was measured using subscales from the diagnostic instrument Mini International Neuropsychiatric Interview 6.0 (MINI; Sheehan et al. 1998). MINI has been validated in several cultural settings and the test re-test reliability of the subscales of relevance have been found to range from 0.76 to 0.93 (Lecrubier et al. 1997). The questions have the response alternatives yes or no, and interviewers follow a manual for assessment. Most questions in MINI concern current issues, therefore, to be able to assess lifetime problems this time frame was added to the questions. Generalized anxiety disorder, panic disorder, social phobia and post-traumatic stress disorder were combined into ‘anxiety disorders’. The information about diagnoses were combined into dichotomous variables (never/ever).

Suicidal ideations and attempts were measured using the suicide questions in the depression subscale. Since those questions were only asked to participants endorsing the criteria for depression, three additional questions about lifetime suicidal thoughts, wishes and plans was added. Those three questions were only asked to participants not answering the original questions regarding suicide in MINI.

Respondents with both previous and current depression were asked if they during at least two weeks did:

- Think about suicide
- Wish they were dead
- Have a suicide plan or
- Make a suicide attempt.

A positive response to any of the three first questions defined the concept suicidal ideations.

The following questions were asked participants with no previous or current depressive episodes (giving 8 of 75 attempts, and 55 of 312 with ideations only), and hence not asked the suicide-related questions connected to those episodes.

During your life-time, have you ever:

- During two weeks or longer, felt that you wanted to die?
- Felt so blue that you considered taking your own life?
- Made a suicide attempt?

A positive response to one of the first two questions was defined as suicidal ideations.

Participants endorsing any of the questions on suicidal ideations were categorized as ‘ideators’, and participants endorsing the question on suicide attempts were categorized as ‘attempters’.

#### Gambling

Swelogs includes two gambling measures: The South Oaks Gambling Screen-Revised Life Time measure (SOGS-R Life) and the Problem Gambling Severity Index (PGSI; Ferris and Wynne 2001). The SOGS was developed to use in clinical settings among adults (Lesieur and Blume 1987). In Swelogs, the SOGS-R was included to enable comparisons with previous studies that have used the instrument, and because it includes life-time problem gambling, which other instruments often do not. The psychometric properties of the instrument have been evaluated with satisfactory results; test re-test reliability 0.71–0.74 and internal consistency 0.97 (Lesieur and Blume 1987; Stinchfield 2002). SOGS-R consists of 21 items, of which 20 dichotomous items adds up into a summary score of 0–20 points. To measure problem gambling in the past twelve months the PGSI was used and it was administered to respondents who had reported any gambling in the past twelve months. The PGSI was developed to measure problem gambling in the general population from a public health perspective, focusing on harm and consequences (Ferris and Wynne 2001). Studies have shown that the PGSI have high internal reliability; 0.85 (Holtgraves 2009; Orford et al. 2010). PGSI consists of 9 items with response alternatives from never to almost always (0–3 points per item), with a maximum score of 27 points. It is recommended that, based on the sum-score, respondents are categorized into: non-problem gambling (0), low-risk gambling (1–2), moderate risk gambling (3–7), and problem gambling (8+) (Ferris and Wynne 2001). In practice, to increase statistical power the categories problem gambling and moderate risk gambling are often collapsed to one. In the Swelogs project, as well as in this study, the categories with a score of 3–7 and 8–27 was collapsed to one category, problem gambling.

#### Other Measures: Socio-Economic Status

Socio-demographic information was gathered from official national registers. The variable socio-economic status was based on educational level and was categorized as follows; low SES-primary or lower secondary school, medium SES-upper secondary school and high SES-post-secondary or tertiary school.

### Response Rate and Attrition

During fall 2011, 1 876 interviews were conducted, giving a response rate of 78.2%. A larger proportion of the controls responded, compared to the cases (87.5% versus 72.3%). There were no differences in response rate across gender.

### Analyses

Cases and controls were compared regarding suicidal ideations and attempts using Chi-square tests. Logistic regression was used to examine the association between lifetime problem gambling and suicidality. To check for multicollinearity, Variance Inflation Factors (VIF) were calculated through linear Regression (Midi et al. 2010) where all variables were included. All VIF-scores were between 1.0 and 1.2 and multicollinearity is therefore not likely to be a problem. Separate analyses were conducted with attempts and ideations (excluding attempters) as dependent variables. Variables known in previous research (Borges and Loera 2010; Franklin et al. 2017; Hawton et al. 2013; Hodgins et al. 2011; Sareen et al. 2005; van der Maas 2016) to be associated with both problem gambling and suicidal events, and admitted for in the Swelogs ID-track, were chosen as confounding variables in the analyses (socio-economic status, alcohol dependence, illicit drug abuse, depression and anxiety). The crude model shows the overall association between problem gambling and suicidal thoughts/attempts. Model 1 was adjusted for socio-economic status. Model 2 was adjusted for socio-economic status, alcohol dependence and illicit drug abuse. Finally, life-time depression and anxiety were added in Model 3. Data was analyzed using IBM SPSS statistics 24.

Since the cases and controls in this study were matched based on age and gender, those variables were not included in the models. Using logistic regression, sensitivity analyses conducted including those variables did not show any significant effect on the overall association between problem gambling and suicidal attempts/ideations. Lifetime measures was used for all psychiatric conditions. Sensitivity analyses was conducted including present psychiatric conditions, as well as lifetime alcohol misuse and lifetime illicit drug use dependence. However, they did not affect the models and were therefore not included.

As common in case-control studies, associations (in this case between problem gambling and suicidality) were estimated using odds ratios. Since case-control studies are typically done when the study outcome is uncommon in the population, odds ratios will approximate risk ratios (Kelsey et al. 1996; MacMahon and Pugh 1970).

Finally, gamblers with and without suicidal behaviours were compared with regard to the above variables using Chi-square tests. Since we hypothesized, based on previous research that financial difficulties might discriminate attempters from non-attempters (Meltzer et al. 2011; Richardson et al. 2013), payment defaults was added in this analysis.

## Results

Suicidal attempts were twice as common among the cases (6.6%) compared to the controls (3.3%) Chi-square = 9.0 (1), *p* = 0.003. Suicidal ideations (attempters excluded) was almost twice as common among the cases (21.2%) compared to the controls (11.2%), Chi-square = 25.2 (1), *p* =  < 0.001.

In the first set of binary logistic regression models, the association between life-time problem gambling and life-time suicidal attempts was examined (controls = ref). As shown in Table 2 there was a bivariate association between problem gambling and suicidal attempts. This association remained significant when controlling for socio-economic status. However, when also controlling for alcohol dependence and illicit drug abuse, the association between problem gambling and suicidal attempts ceased to be significant. Lifetime illicit drug abuse, and lifetime depression had the strongest impact on the association.

**Table 2:** Comparison between cases and controls regarding the association between problem gambling and: 1. suicide attempts and 2. suicidal ideations (N = 1859)

	Crude Model OR (CI 95%)	Model 1 OR (CI 95%)	Model 2 OR (CI 95%)	Model 3 OR (CI 95%)
1. Attempts (n = 74)				
Problem gambling (controls ref)	2.1 (1.3–3.4)	2.1 (1.2–3.4)	1.6 (0.9–2.8)	1.2 (0.7–2.2)
SES low vs high (ref)		2.6 (1.4–5.0)	2.5 (1.3–4.8)	2.2 (1.1–4.4)
Alcohol dependence			2.5 (1.3–5.0)	1.7 (0.8–3.6)
Illicit drug abuse			6.8 (3.2–14.3)	4.1 (1.8–9.4)
Anxiety				2.5 (1.4–4.3)
Depression				11.0 (5.3–22.8)
2. Ideations (n = 228)				
Problem gambling (controls ref) (controls = ref)	2.1 (1.6–2.8)	2.2 (1.6–2.9)	1.9 (1.4–2.6)	1.5 (1.1–2.1)
SES low vs. high (ref)		1.7 (1.2–2.3)	1.6 (1.2–2.3)	1.6 (1.1–2.4)
Alcohol dependence			1.9 (1.3–2.9)	1.4 (0.9–2.2)
Illicit drug abuse			4.5 (2.6–7.8)	3.2 (1.7–6.0)
Anxiety				2.3 (1.6–3.2)
Depression				7.3 (5.3–9.9)

Crude Model: Unadjusted

Model 1: Adjusted for socio-economic status (SES)

Model 2: Adjusted for socio-economic status (SES), lifetime alcohol dependence and lifetime illicit drug abuse

Model 3: Adjusted for socio-economic status (SES), lifetime alcohol dependence, lifetime illicit drug abuse, lifetime anxiety and depression

In the second set of binary logistic regression models, the association between problem gambling and suicidal ideations was studied (see Table 2). Like with suicidal attempts, the risk for having had suicidal ideations were more than double for the cases compared to the controls, and the association remained significant after controlling for socio-economic status. As opposed to the models on suicidal attempts, the association between problem gambling and suicidal ideations remained significant after controlling for lifetime alcohol dependence and illicit drug abuse. The association also remained significant in the full model, when lifetime anxiety and depression was added.

In sum, problem gambling seems to be directly associated with suicidal ideations, but not with suicide attempts, after controlling for other psychiatric conditions.

Finally, problem gamblers (the cases) with and without lifetime suicide attempts/ideations were compared. As shown in Table 3, attempters differed significantly on all variables, except problem gambling severity, compared to non-attempters. More attempters, compared to non-attempters, had low socio-economic status, had payment defaults, and had suffered from alcohol dependence, illicit drug abuse, anxiety and depression.

**Table 3:** Comparison of attempters versus non-attempters and ideators vs, non-ideators

Variables	Non- attempters (n = 368)	Attempters (n = 26)	Chi-square	Df	*p*	Non-ideators (n = 298)	Ideators (n = 78)	Chi-square	Df	*p*
Low SES	24.3	53.8	10.9	2	.004	22.7	30.3	7.4	2	.025
Alc. dependence	19.3	46.2	10.5	1	.003	18.3	23.1	.88	1	.216
Illicit drug abuse	4.6	23.1	15.0	1	.002	3.5	9.0	4.2	1	.047
Anxiety	21.0	46.2	8.8	1	.005	16.0	40.0	20.6	1	< .001
Depression	29.3	84.6	33.6	1	< .001	18.8	67.9	71.5	1	< .001
PGSI 8 +	3.9	7.9	1.5	1	.195	17.0	31.6	5.5	1	0.22
Payment defaults	4.4	16.2	21.54	1	< .001	3.8	8.0	8.62	1	.006

The same pattern was found among participants with previous suicidal ideations (but no actual attempts) compared no non-ideators, however only anxiety and depression and payment defaults differed significantly.

Interestingly, having had payment defaults was almost four times as common among attempters compare to non-attempters. Also, lifetime illicit drug abuse were more than four times as prevalent among the attempters. Gambling severity (PGSI 8+), was the factor that yielded the smallest difference between attempters and non-attempters. For ideators, the largest difference compared to non-ideators were on lifetime depression, whereas alcohol dependence generated the smallest difference.

## Discussion

In this study, the associations between problem gambling and suicidal ideations/attempts was examined using a case control design with a sample from the general Swedish population. Both ideations and attempts were about twice as frequent among the cases compared to the controls. After controlling for socio-economic status and life-time mental health problems, rates of lifetime suicidal ideation (thoughts and plans) was significantly higher among the cases compared to the controls. The association between problem gambling and lifetime attempts ceased to be statistically significant when employing the same covariates as mentioned above.

When comparing cases with and without suicide attempts/ideations, the largest difference between attempters and non-attempters were on payment defaults and illicit drug abuse, whereas depression yielded the largest difference between ideators and non-ideators. Problem gambling severity (PGSI 8+) resulted in the smallest difference, compared to the other variables, between attempters and non-attempters.

The results from this study suggests (even thought there were small differences) that problem gambling on its own may have an effect on suicidal ideations, whereas for an actual suicide attempt to happen, additional factors might be needed. Financial difficulties and illicit drug abuse were such factors that might have played that role among the cases in this study.

The findings from this study are in line with the findings from the study by Thomsson and Newman (2005), where the association between problem gambling and suicide attempts ceased to be statistically significant after controlling for mental illness. On the other hand, in our sample, problem gambling was associated with suicidal ideations even when considering other psychiatric conditions.

The association between problem gambling and suicidal behavior (ideations and attempts) were weaker in this study than in some previous works (Battersby et al. 2006; Newman and Thompson 2007; Petry and Kiluk 2002) and this might be due to the non-clinical sample from the general population, as well as the low threshold for problem gambling used in this study.

The finding that even sub-clinical levels of problematic gambling also seem to increase the risk of suicidal ideations is important information that ads to the existing literature. This fact, that the groups of predominantly subclinical gamblers in this study showed an increased risk of suicidal ideations emphasize that risk gambling and problem gambling that does not fulfill the requisites for a diagnosis also deserves special attention from a preventive perspective.

Having said that, it seems that mild problem gambling per se might not be enough for progressing from suicidal ideations to actual attempts unless combined with other psychiatric comorbidity such as depression or substance use problems, or other factors such as financial difficulties. On the other hand, it is well known than the co-occurrence of psychiatric comorbidity is high among problem gamblers (Lorains et al. 2011). Especially substance use disorders, depression and anxiety disorders are commonly found in problem gamblers, with a prevalence rate of about 58%, 38% and 37% respectively. With that in mind, when clinicians meet problem gamblers, suicide ideations and previous attempts should be explored.

A major strength in this study is the use of a sample from the general population, which generates more generalizable results compared to results from studies using treatment seeking samples. Another strength is that psychiatric diagnoses were based on clinical interviews rather than self-assessment measures. Yet another strength is the inclusion of problem gamblers ranging in severity from mild to severe, mirroring the actual gambling situation in society. In addition, the distinction between suicidal ideations and attempts make it possible to find divergent patterns.

Some of the psychiatric factors used as covariates in this study are reciprocally interwoven. Hence, establishing causality in this type of study is certainly difficult. Our analyses are based on some assumptions regarding causality. For instance, we assume that mental health issues precede suicidal behaviors. Some previous research seem to confirm this (Miché et al. 2018; Sundqvist and Rosendahl 2019). Having said that, the mental health variables refer to lifetime problems, which makes it impossible to pinpoint these events in time, which is a limitation of the study.

More of the risk factors that lead to suicidal ideations does not necessarily lead to suicidal attempts. Instead, progression from suicidal ideations to attempts might require additional factors. Hence, a suggestion for future research concern factors that discriminate between suicidal ideations and suicidal attempts among problem gamblers. Further, the fact that problem gambling, mental health issues and suicidal behaviours appear in different cultural settings, calls for more population-based studies on this topic.

## Conclusions

Problem gambling is associated with suicidal ideations and attempts in the general population, even when including sub-clinical levels of problem gambling. However, the association between problem gambling and suicidal attempts is in part explained by other mental health variables (depression, anxiety, AUD and SUD). Even though no conclusion regarding the casual relationship can be drawn in this type of study, it seems like sub-clinical levels of problem gambling might have an impact on suicidal ideations whereas for suicide attempts to occur, other factors need to be present. Payment defaults and illicit drug abuse may be example of such factors.
